# Introducing hat graphs

**DOI:** 10.1186/s41235-019-0182-3

**Published:** 2019-08-14

**Authors:** Jessica K. Witt

**Affiliations:** Department of Psychology, Colorado State University, Fort Collins, CO 80523 USA

**Keywords:** Information visualization, Graphs

## Abstract

Visualizing data through graphs can be an effective way to communicate one’s results. A ubiquitous graph and common technique to communicate behavioral data is the bar graph. The bar graph was first invented in 1786 and little has changed in its format. Here, a replacement for the bar graph is proposed. The new format, called a hat graph, maintains some of the critical features of the bar graph such as its discrete elements, but eliminates redundancies that are problematic when the baseline is not at zero. Hat graphs also include design elements based on Gestalt principles of grouping and graph design principles. The effectiveness of the hat graph was tested in five empirical studies. Participants were nearly 40% faster to find and identify the condition that led to the biggest difference from baseline to final test when the data were plotted with hat graphs than with bar graphs. Participants were also more sensitive to the magnitude of an effect plotted with a hat graph compared with a bar graph that was restricted to having its baseline at zero. The recommendation is to use hat graphs when plotting data from discrete categories.

## Significance

Visualizations are an important way to communicate results from data. As “big data” has increased impact on daily life, communication of data is of critical importance. The bar graph is ubiquitous and yet has not been fundamentally updated since its inception in the eighteenth century. The hat graph is offered as a modernized version that more effectively communicates differences across conditions. Hat graphs increased the speed to find the condition associated with the biggest difference by nearly 40% relative to bar graphs. Hat graphs also increased sensitivity to the size of an effect by 30% and eliminated bias in estimating effect size. Hat graphs can significantly improve how scientists communicate their data.

## Introducing hat graphs

The bar graph is commonly used to visualize data in psychology. In a recent issue of *Psychological Science* (2018, v 29, issue 12), 50% of the articles included a bar graph. The bar graph dates back to 1786 (Playfair, 1786), and the first bar graph bears great resemblance to typical bar graphs used today. Bar graphs are problematic in presenting results from behavioral research. Bar graphs depict values in two ways: one is by the relative position of the end of the bar and the other is by length of the bar (and, perhaps a third, is by area of the bar). These various sources of information will be inconsistent with each other if the baseline of the graph is not set to zero. When comparing conditions represented by separate bars, the relative position of the ends of the bars will accurately reflect the differences between the conditions, but the relative difference in length will exaggerate the differences (Healy, 2019; Pandey, Rall, Satterthwaite, Nov, & Bertini, 2015; Pennington & Tuttle, 2009). Thus, the rule for bar graphs is to always set the baseline to zero. Of the articles that used bar graphs referenced above, all but one used zero as the baseline. However, even a baseline at zero creates large biases in readers’ perceptions of the size of effects depicted in bar graphs: big effects can appear small with a baseline at zero (Witt, in press). One way to improve readers’ perceptions is to maximize compatibility between the visual size of the effect and the effect size being depicted. Given that effect size in psychology is often measured in terms of SDs, and an effect size of 0.8 is considered “big” (Cohen, 1988), it is sensible to set the range of the y-axis to 1.5 SDs. With this range, big effects look big and small effects look small (Witt, in press). This range is problematic when using bar graphs, however, given the mixed meanings across the different features when the baseline is not set to zero.

Alternatives to bar graphs include point graphs and line graphs. Point graphs have the advantage that only one feature specifies the data, namely relative position. Thus, inconsistencies are not created by non-zero baselines. But with point graphs the Gestalt grouping principles to help facilitate perceptual grouping of pairs of data points are not as strong. This grouping problem can be solved by connecting the points with a line, thus making the graph a line graph. Line graphs are a natural choice when communicating trends, such as differences in a dependent variable across a continuous independent variable, because a feature of the line (the slope) represents the trend, without having to integrate across multiple features (Carswell & Wickens, 1996). Line graphs are not, however, a natural choice when communicating discrete values. They can even lead to misinterpretations of discrete variables as continuous. For example, when comparing across distinct groups like construction workers versus librarians, people were more likely to make continuous comparisons like “the more librarian a person is, the shorter he is” rather than discrete comparisons like “librarians tend to be shorter than construction workers” when presented with line graphs than when presented with bar graphs (Zacks & Tversky, 1999). Lines are also an excellent choice for communicating interactions because the interactions are represented by the intersection in the lines, so the interaction can be perceived by comparison across the slopes of the lines, rather than integrating across four or more bars. Some have recommended that line graphs be used to display interactions even across discrete categories (Kosslyn, 2006).

Rather than have to select between these various trade-offs between bar and line graphs, another option is to design a new kind of graph that has the desirable properties of the bar graphs (proper interpretation of discrete categories) and desirable properties of the line graphs (configurable properties that signal the effect of interest, unrestricted settings for the y-axis). Often the purpose of a graph is to report findings of a difference between two conditions (a main effect) or a difference between differences in conditions (an interaction). Bar graphs are not the most effective or efficient way to communicate differences because they require additional processing. According to Pinker’s theory of graph comprehension, objects give rise to “message flags” that make the objects’ values “easily extractable” from the graph (Pinker, 1990, p. 108). For bar graphs, each bar has an associated message flag to signal its height but extracting the difference across two bars (or the differences across two pairs of bars in the case of an interaction) requires additional processes of what Pinker refers to as interrogation. In the case of bar graphs, this will require top-down visual search processes to locate the relevant bars and then mentally compare their relative heights. A better way to communicate differences is to represent the difference as a single object. Thus, the difference would have its own message flag automatically associated with it, rather than require these additional interrogation processes.

**Fig. 1:**
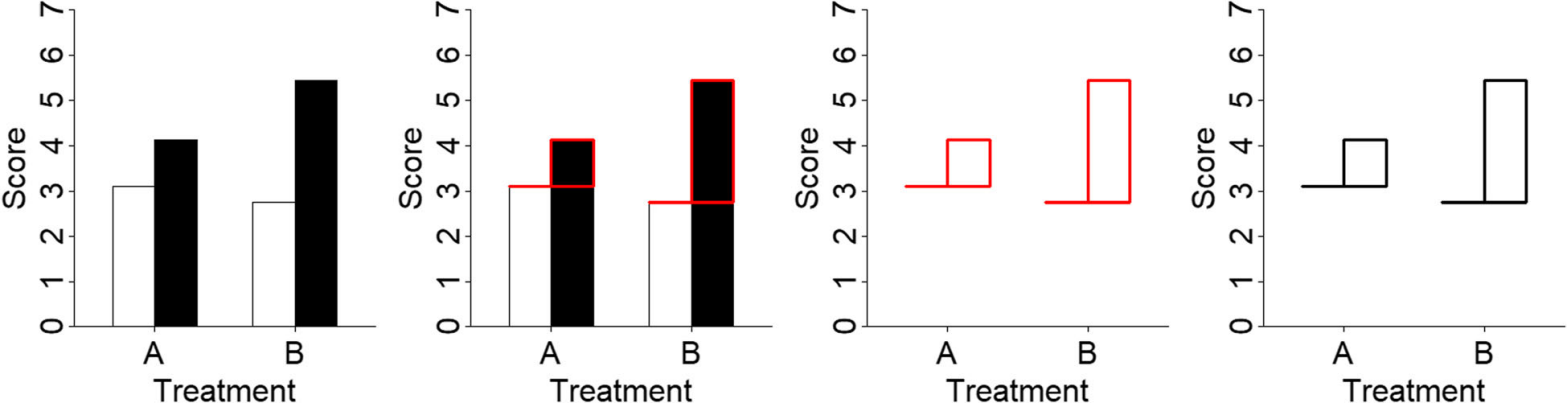
Illustration of the format of the hat graph as it relates to a bar graph

To achieve these objectives, the traditional bar graph was transformed. First, the tops of the bars were retained while the bars themselves were removed. This removes the redundancy between specifying the values by the tops of the bars and by the length of the bars. Removing redundancy is one of the recommendations made by Tufte (2001), and by removing bar length as a signifier of value, the y-axis does not have to start at zero because now the tops are the only indicator of value and not also bar length. Second, the difference between two sets of bars was highlighted by enclosing this difference as its own object by keeping the portion of the second bar that *differed* from the first bar (see Fig. 1). Third, the components directly abutted each other in order to evoke strong Gestalt principles of grouping, namely connectedness and proximity. The new format is called a *hat graph* because the graphs ended up bearing a resemblance to hats. The “brim” of the hat represents the value for condition 1, and the top of the “crown” of the hat represents the value for condition 2. The height of the crown represents the difference. A single object (the crown) represents the difference, so it should be easier and faster to see the differences represented in the graph. This prediction was tested in experiments 1 and 2.

A second prediction was that hat graphs would lead to better sensitivity and less bias in estimating the magnitude of the effect. This prediction was based on the idea that hat graphs allow for more flexibility in setting the range of the y-axis, and that setting the y-axis range to 1.5 SDs, as recommended by Witt (in press), improves sensitivity and decreases bias relative to showing the full range. This prediction was tested in experiment 3.

## Experiment 1

Participants were shown images depicting attitude scores on baseline and final tests for three or six advertisements. Their task was to indicate which advertisement produced the largest improvement in attitude at final score over baseline.

### Method

#### Participants

Twenty-two participants volunteered in exchange for course credit. A large effect was assumed, given the theoretical reasons to think that hat graphs would have an advantage over bar graphs. A power analysis for a paired-samples *t* test with an effect size of *d* = 0.80 and alpha = 0.05 (two-tailed) showed that 14 pairs are needed to achieve 80% power. Data collection was scheduled to stop on a day on which this number was likely to be achieved, although more participants were collected than needed, resulting in 95% power to find an effect size of *d* = 0.80.

#### Stimuli and apparatus

**Fig. 2:**
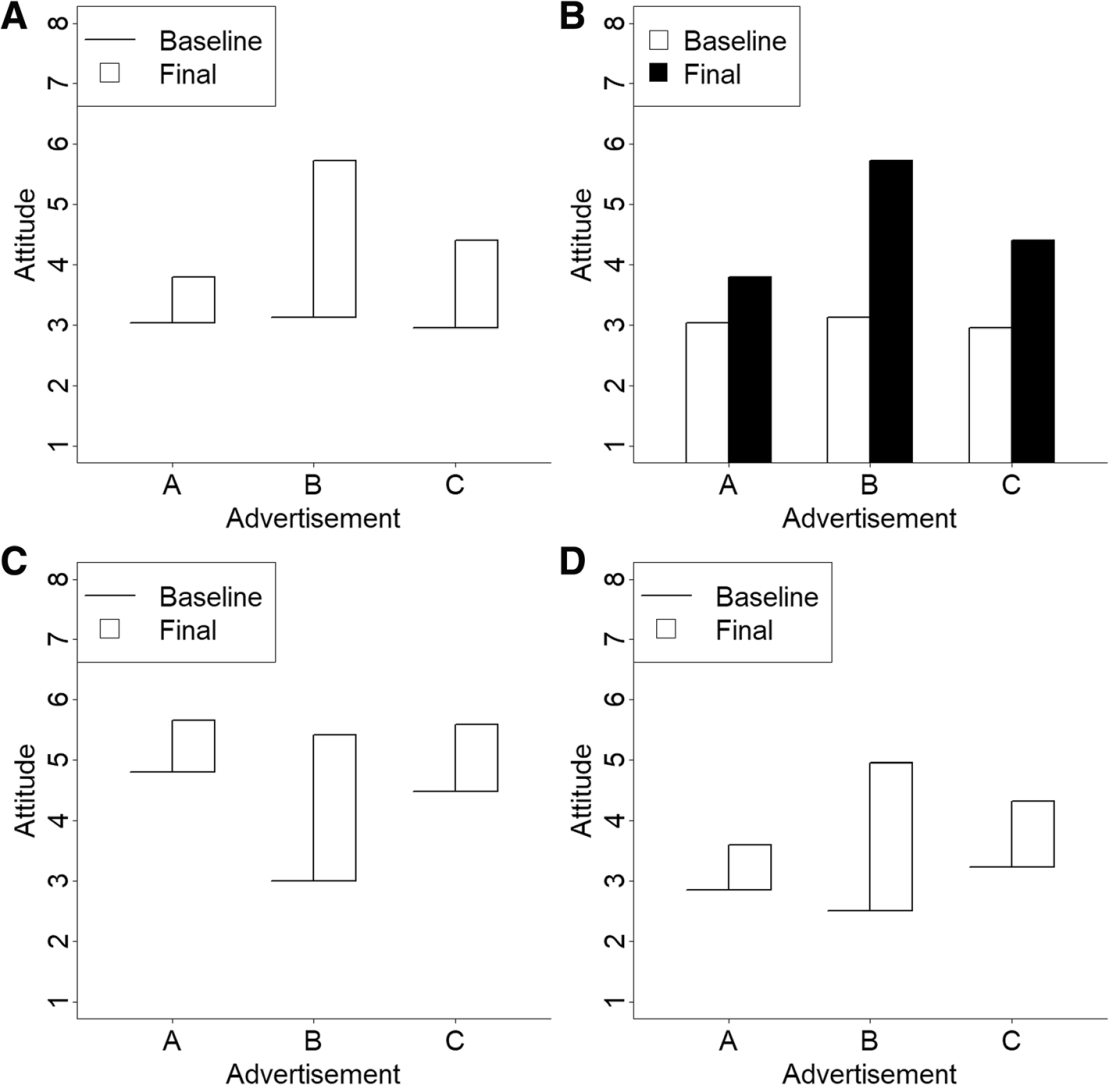
Sample stimuli from experiment 1. Panels **a** and **b** show the hat graph version and the bar graph version of the same data. Panels **a**, **c**, and **d** show the three types of alignment (baseline, final, mean, respectively)

Stimuli were displayed on computer monitors. The stimuli were created using data simulated and plotted in R (R Core Team, 2017). Four factors were manipulated. One factor was graph type (hat graph versus bar graph). Each set of simulated data were plotted with a hat graph and with a bar graph. Another factor was number of advertisements (three or six). A third factor was the position of the target (best) advertisement. These were evenly distributed across the locations, and target position was repeated for the graphs with only three advertisements. The fourth factor was the alignment across advertisements. One third of the graphs were aligned to have similar baselines, so the target advertisement also had the highest final score. One third were aligned to have similar final scores, so the target advertisement had the lowest baseline score. And one third were aligned at the mean value between baseline and final scores (see Fig. 2). Each graph style was repeated four times to have several variants to show to the participants. This resulted in 288 unique graphs (288 = 2 × 2 × 6 × 3 × 4).

Data for the graphs were created from simulations. As noted below, the task for participants was to indicate which advertisement produced the largest *change* in attitude, so the critical data are the differences between conditions. For the non-target advertisements, the differences between baseline and final scores were the mean value of 100 samples from a normal distribution with a mean of 1 and a SD of 2. For the target advertisements, the differences between baseline and final scores were the mean value of 100 samples from a normal distribution with a mean of 2.5 and a SD of 1. Thus, the target advertisement produced a bump in attitude scores 2.5 times more than the non-target advertisements. These difference scores were added to the baseline scores for the baseline aligned and mean aligned graphs. For these graphs, the baseline scores were the mean value of 100 samples from a normal distribution with a mean of 3 and a SD of 1. For the final-align graphs, the final scores were the mean value of 100 samples from a normal distribution with a mean of 5.5 and a SD of 1, and the difference scores were subtracted from the final scores to compute baseline scores. The process was the same for the target conditions with the exception that 0.5 was subtracted from the baseline condition so that it would not align with the other baseline conditions.

Two graphs were created for each set of data. One was a bar graph and one was a hat graph. Thus, the data contained in the graphs were identical across graph types. The baseline condition was white and the final condition was black for the bar graph. The lines were black and the crown was white for the hat graphs. The y-axis ranged from 1 to 8 on every graph. Advertisements were labeled A–F and were always in alphabetical order.

#### Procedure

Participants completed two blocks of trials, one with bar graphs and one with hat graphs. Start order was counterbalanced across participants. For the hat graphs, they were shown these initial instructions: “An advertising company is interested in which ads lead to the biggest changes in attitude. They ran a study testing several different ads. In each study, they measured attitude at BASELINE (before seeing any ads) and again at the FINAL test (after seeing the ads). All of the ads increased attitude. Your task is to determine which ad produced the BIGGEST increase in attitude. The baseline attitude will be shown as a horizontal line. The final attitude is shown as the top of the box. The height of the box shows the change in attitude from baseline to final test. Which ad produces the biggest change? Enter your response for each graph on the keyboard. Respond as fast and accurately as possible. Press ENTER to begin”. For the bar graphs, the instructions were the same except instead of describing the hat graph, they were told the following: “The baseline attitude will be shown in white boxes. The final attitude will be shown in black boxes. The difference between the white and black boxes shows change in attitude from baseline to final test.”

On each trial, a graph was shown after a fixation screen of 500 ms, and participants entered a response A–C (for graphs with three advertisements) or A–F (for graphs with six advertisements). The graph remained visible until participants made their response, at which point a blank screen was shown for 500 ms before the next trial began. Participants completed 144 trials with one type of graph before switching to the block of trials with the other graph type. Order within block was randomized.

### Results and discussion

Reaction times (RTs) are positively skewed, so they were log-transformed. The data were initially explored for outliers. RTs beyond 1.5 times the interquartile range (IQR) for each subject for each condition were excluded (3% of the data). Next, mean RTs and mean accuracy scores were calculated for each subject and each condition and plotted in separate boxplots. One participant was beyond the IQR for both, and three participants were beyond 1.5 times the IQR for accuracy scores. These participants were excluded. For remaining participants, accuracy was nearly perfect (mean (M) = 98.9%, SD = 1.3%), so the analysis focused on RTs.

Data were analyzed with linear mixed models using the lme4 and lmerTest packages in R (Bates, Machler, Bolker, & Walker, 2015; Kuznetsova, Brockhoff, & Christensen, 2017). A linear mixed model was run with the log RTs as the dependent factor. The independent factors were graph type (bar or hat), number of advertisers (three or six), graph alignment (baseline, final, mean), and initial graph type (bar or hat). All independent factors were entered as a factor with the reference factor being the first as listed above. Two-way interactions between graph type and each factor were also included. The random effects for participant included intercepts and slopes associated with graph type. Estimation was done using restricted maximum likelihood and Satterthwaite’s method for degrees of freedom. Effect sizes were calculated based on the formula from Westfall, Kenny, and Judd (2014). The emmeans R package was used to extract marginal means on the original scale (non-transformed RTs) from the model for the plots (Lenth, 2019).

**Fig. 3:**
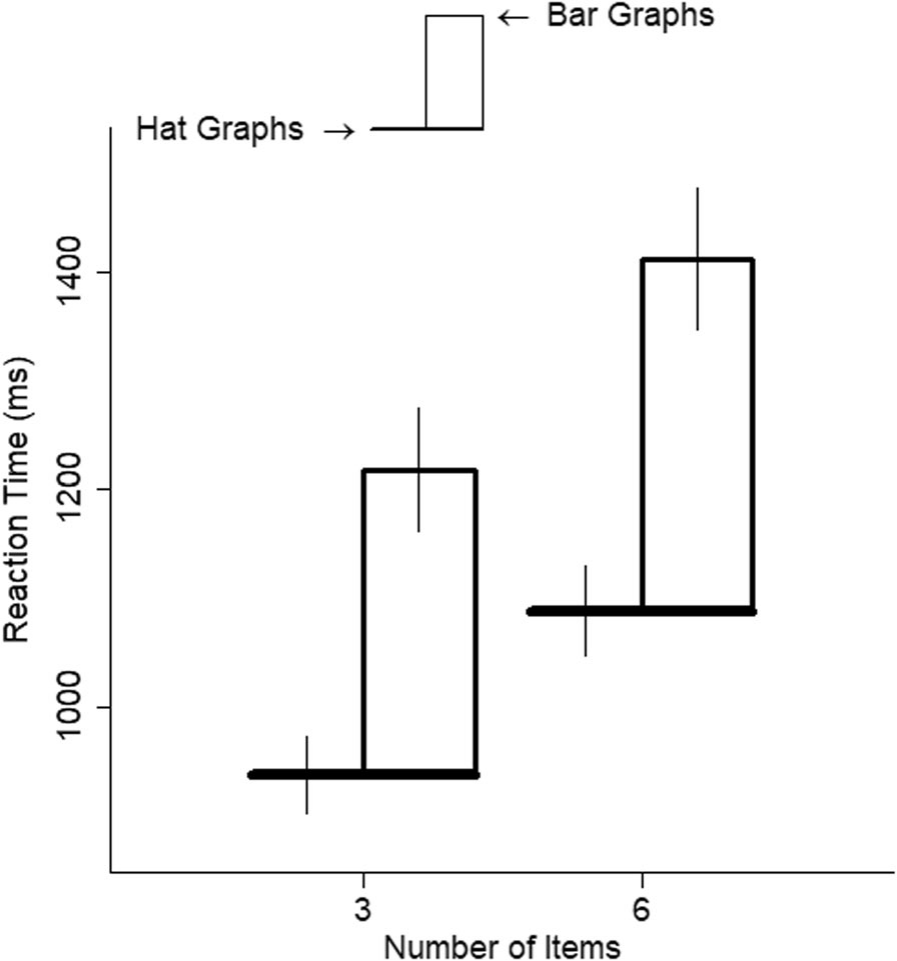
Reaction time (RT) is plotted as a function of number of items and graph type for experiment 1. The brim of the hat corresponds to the RTs for the hat graph condition, and the top of the crown of the hat corresponds to RTs for the bar graph condition. The size of the crown indicates the difference in RTs between the two conditions. Error bars are ±1 SEM, estimated from the model

**Fig. 4:**
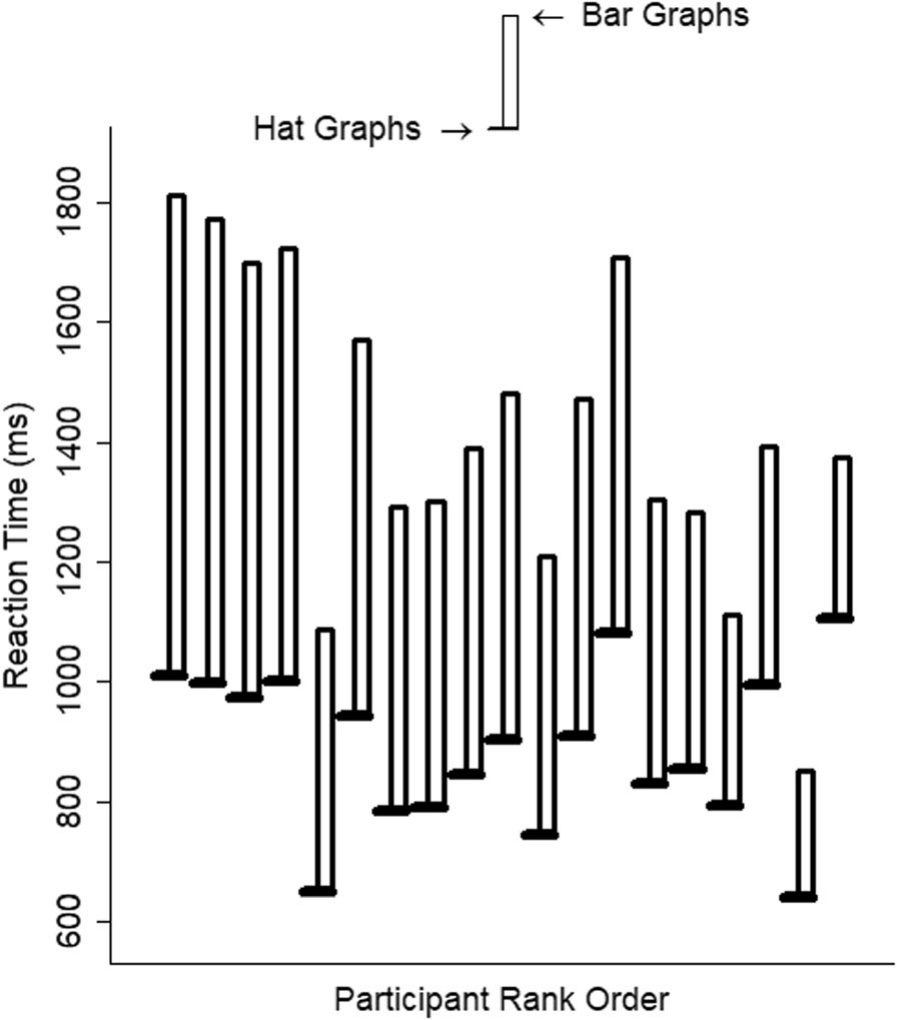
Estimated reaction time (RT) is plotted as a function of graph type and participant for experiment 1. Estimates are based on the random intercepts and slopes. The brim of the hat corresponds to the RTs for the hat graph condition, and the top of the crown of the hat corresponds to RTs for the bar graph condition. The size of the crown indicates the difference in RTs between the two conditions

Graph type had a large effect on RTs, *d* = 1.20, *t* = 11.76, *p* < .001. Relative to bar graphs, responses to hat graphs were 37% faster (see Fig. 3). Using the random effect coefficients to estimate the impact of graph type on each participant, it can be seen that the model estimated that all 18 participants showed faster responses to hat graphs than to bar graphs (see Fig. 4).

The number of items had a small-to-medium effect on RTs, *d* = 0.39, *t* = 15.05, *p* < .001. Going from three to six items was associated with a 16% increase in RT (see Fig. 3). The interaction between number of items and graph type was negligible, *d* = 0, *t* = 0.09, *p* > .92. Thus, although hat graphs increased speed to find the largest difference, they did not make the search more efficient, as would have been shown by a shallower slope for the hat graphs.

**Fig. 5:**
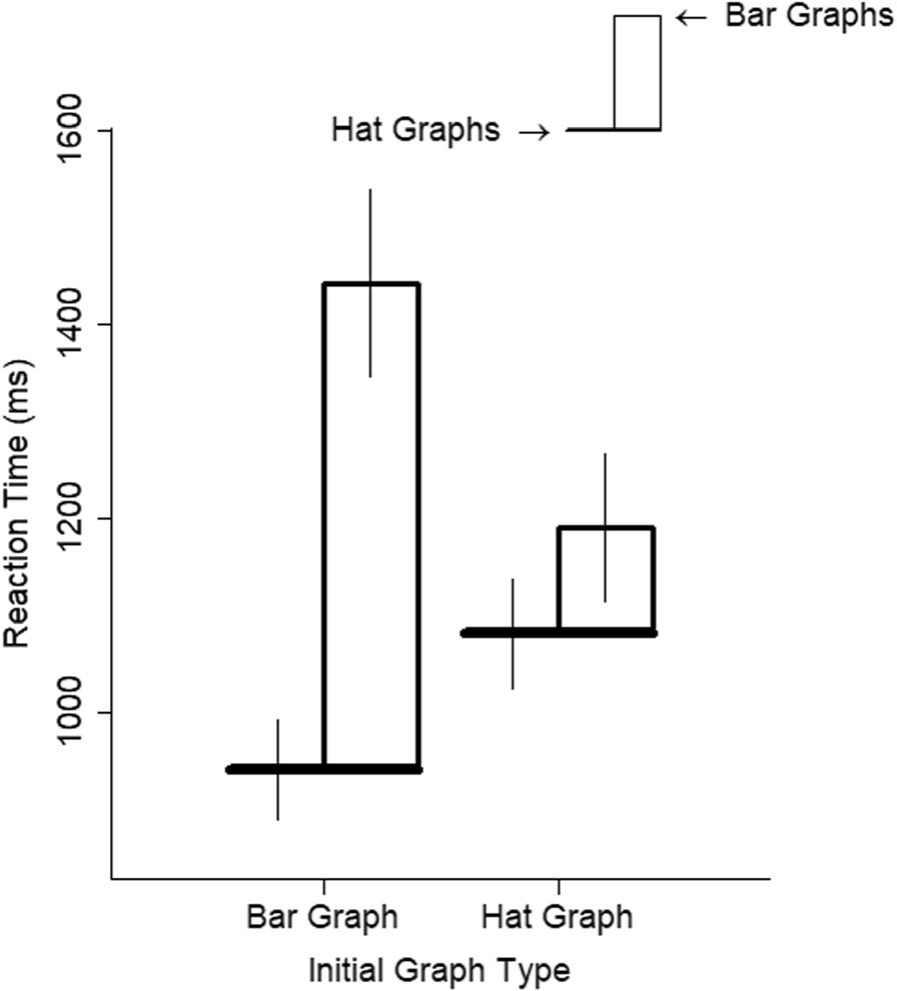
Reaction time (RT) is plotted as a function of graph type and start condition for experiment 1. The brim of the hat corresponds to the RTs for the hat graph condition, and the top of the crown of the hat corresponds to RTs for the bar graph condition. The size of the crown indicates the difference in RTs between the two conditions. Error bars are ±1 SEM based on estimates from the linear mixed model

The medium effect of initial graph type on RTs (*d* = 0.50, *t* = 2.07, *p* = .054) is better explained by the big interaction with graph type, *d* = 0.87, *t* = 6.47, *p* < .001. The increased speed to respond to hat graphs was greater in people who had completed a block of trials with the bar graphs than in those who started with the hat graphs (see Fig. 5). This interaction could also be interpreted as two main effects: faster responses for hat graphs and faster responses in the second block.

**Fig. 6:**
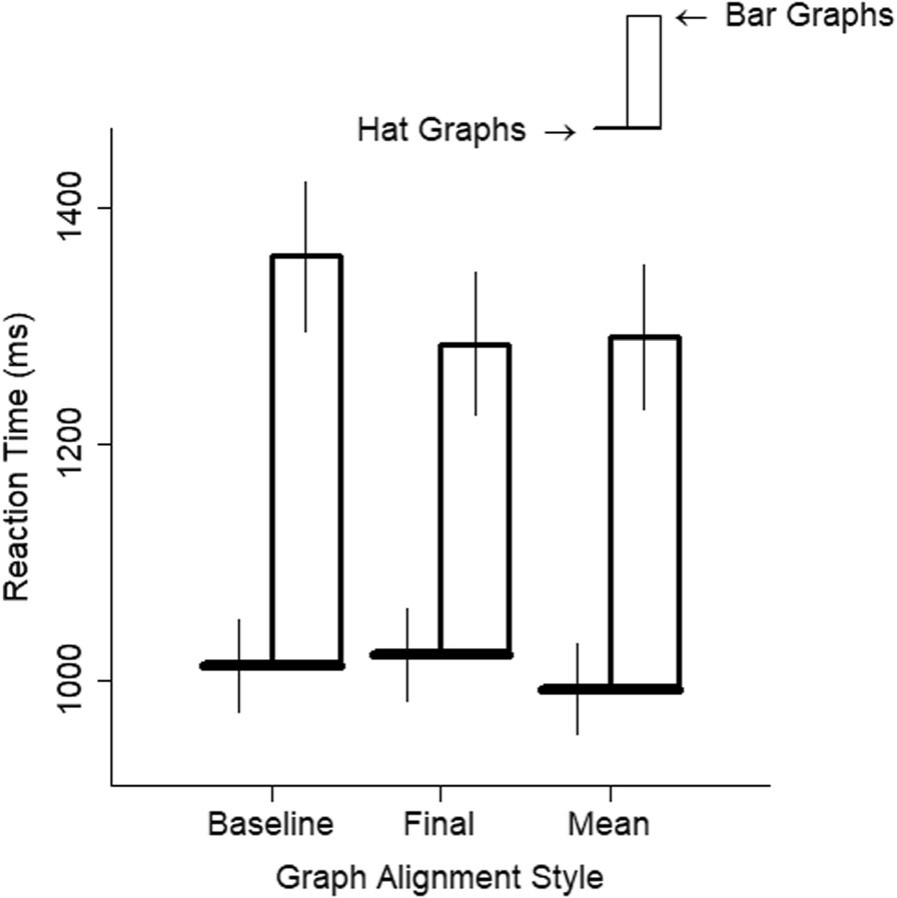
Reaction time (RT) is plotted as a function of graph alignment style and graph type for experiment 1. The brim of the hat corresponds to the RTs for the hat graph condition, and the top of the crown of the hat corresponds to RTs for the bar graph condition. The size of the crown indicates the difference in RTs between the two conditions. Error bars are ±1 SEM estimated from the mixed linear models

Graph alignment had a very small influence on RTs (Fig. 6). The RT was slightly slower when the baseline scores were aligned than when the final scores were aligned, *d* = 0.15, *t* = 4.62, *p* < .001, and slightly slower for baseline scores than when the mean scores were aligned, *d* = 0.13, *t* = 4.26, *p* < .001. There was no difference between RTs for graphs with aligned final scores and aligned mean scores, *d* = 0.01, *t* = 0.36, *p* = .72. The interaction between graph alignment and graph type was similarly quite small (graph type and baseline versus final, *d* = 0.17, *t* = 3.82, *p* < .001; graph type and baseline versus mean. *d* = 0.08, *t* = 1.84, *p* = .066; graph type and final versus mean, *d* = 0.09, *t* = 1.98, *p* = .048). More importantly, responses were faster to hat graphs than to bar graphs in all alignment conditions, *d*s ≥ 0.99, *p*s < .001. This shows some robustness to the advantage of hat graphs over bar graphs because it shows the advantage for hat graphs does not depend on one particular alignment between its parts.

Hat graphs improved speed to find the advertisement that produced the biggest boost in attitude relative to bar graphs. The results are consistent with principles of graph design that by making the difference an object that will give rise to a message flag, graph comprehension will be easier and thus faster.

## Experiment 2

Experiment 2 serves as a replication of experiment 1. The effect size depicted in the graphs was slightly smaller than in experiment 1.

### Method

Seventeen students volunteered in exchange for course credit. Everything was the same as in experiment 1 except the target difference was simulated as a magnitude of 2 (rather than 2.5).

### Results and discussion

Data were analyzed as before and 3% of trials were excluded because the (log) RT was beyond 1.5 times the IQR for that participant for that condition. Mean RTs and proportion of correct scores were summarized for each participant for each condition. Nine participants were identified as outliers based on these summary scores, which seems like a large portion of the data. Statistical models were conducted with all participants, without RT outliers (*n* = 4), without accuracy outliers (*n* = 5), without either RT or accuracy outliers (*n* = 9), and without participants with accuracy scores less than 0.9 (*n* = 3). The outcomes from the various statistical models were the same regardless of which outliers were excluded with the exceptions of the main effect of graph alignment style and the interaction between graph alignment style and graph type for baseline versus final alignments. That the findings are generally the same speaks to the robustness of the effect of graph style, given that outlier removal did not have much influence. The final model reported was one for which the three participants with accuracy scores < 0.9 were eliminated because it seems reasonable that participants who had accuracy scores of 0.7 were different from the participants who had accuracy scores > 0.9 (M = 0.98, SD = 0.03).

The data were analyzed in a linear mixed model with the log of the RTs as the dependent factor and graph type, number of items, graph alignment style, and starting graph type as independent factors. Two-way interactions with graph type and each of the other factors were included. Random effects for participants included the intercepts and slopes for graph type.

**Fig. 7:**
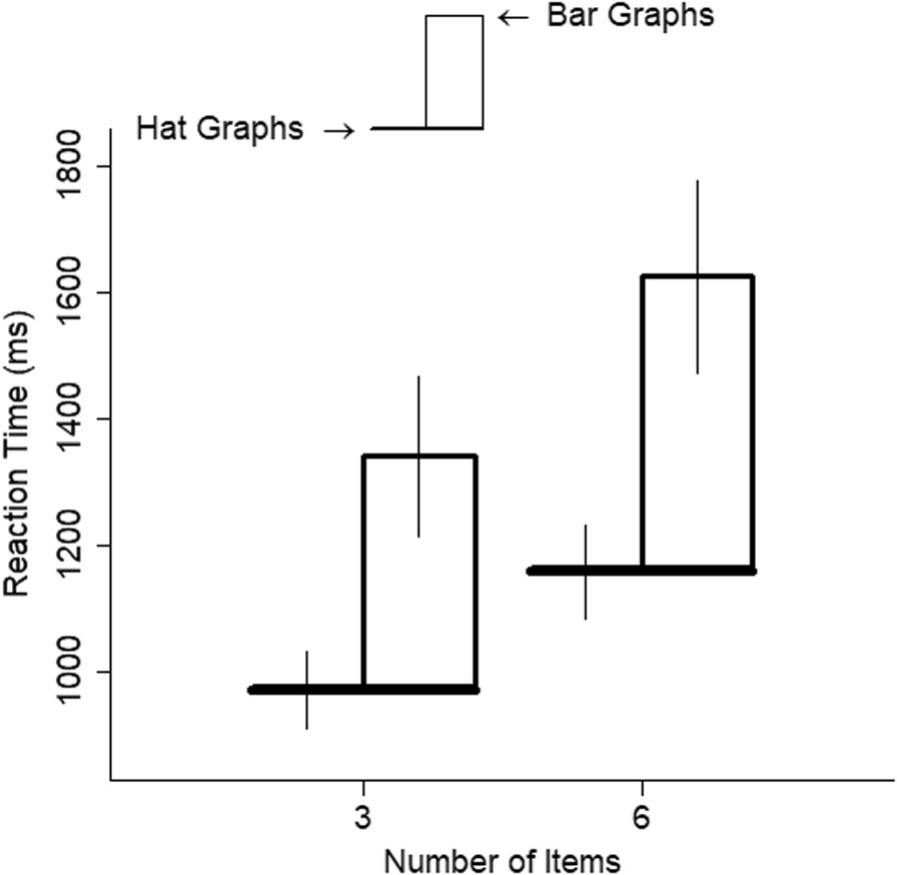
Reaction time (RT) is plotted as a function of number of items and graph type for experiment 2. The brim of the hat corresponds to the RTs for the hat graph condition, and the top of the crown of the hat corresponds to RTs for the bar graph condition. The size of the crown indicates the difference in RTs between the two conditions. Error bars are ±1 SEM estimated from the linear mixed model

**Fig. 8:**
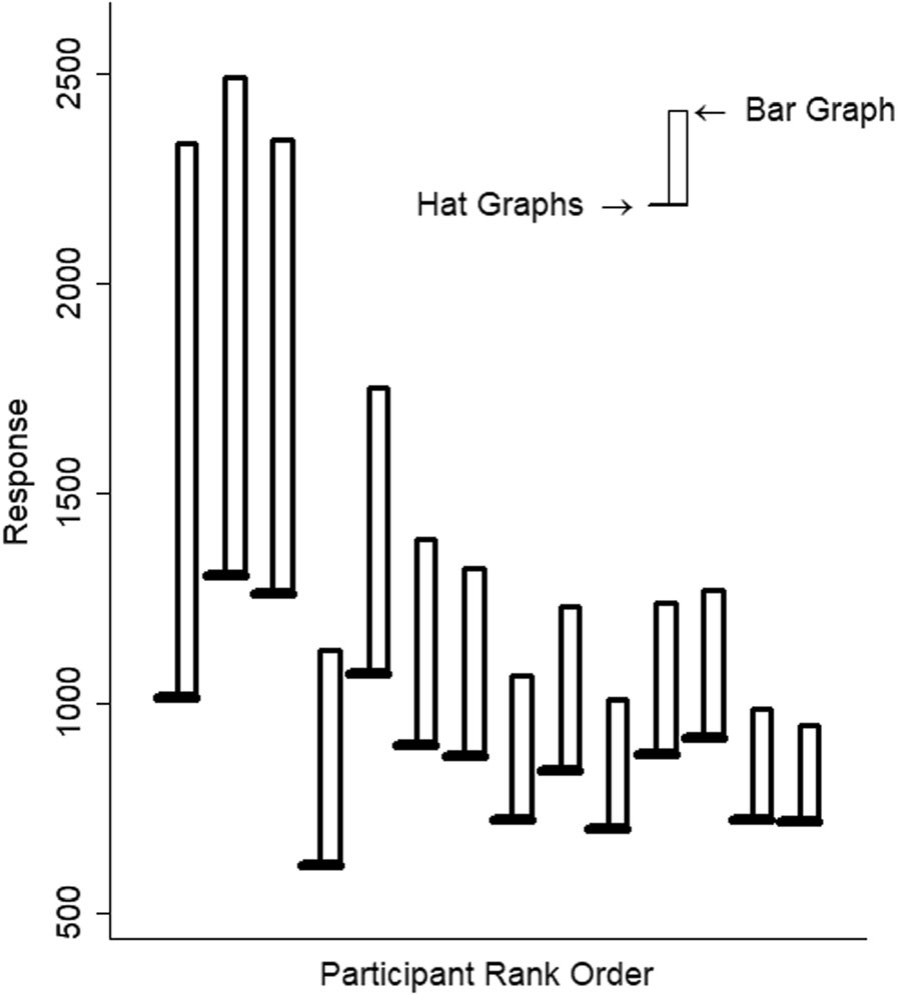
Reaction time (RT) is plotted as a function of graph type and participant for experiment 2. Estimates are based on the random intercepts and slopes from the linear mixed model. The brim of the hat corresponds to the RTs for the hat graph condition, and the top of the crown of the hat corresponds to RTs for the bar graph condition. The size of the crown indicates the difference in RTs between the two conditions

Graph type had a large effect on reaction time, *d* = 0.90, *t* = 6.82, *p* < .001 (see Fig. 7). Participants were 37% faster to respond to hat graphs than to bar graphs. All participants’ responses showed a benefit for hat graphs over bar graphs (see Fig. 8). The number of items had a small effect on reaction time, *d* = 0.34, *t* = 14.77, *p* < .001. Participants were 21% slower to respond to six items than to three items. The interaction between graph type and number of items was negligible, *d* = 0.03, *t* = 0.84, *p* = .40. Thus, although hat graphs increased speed to find the largest difference, it did not make the search more efficient, as would have been shown by a shallower slope for the hat graphs relative to the bar graphs.

**Fig. 9:**
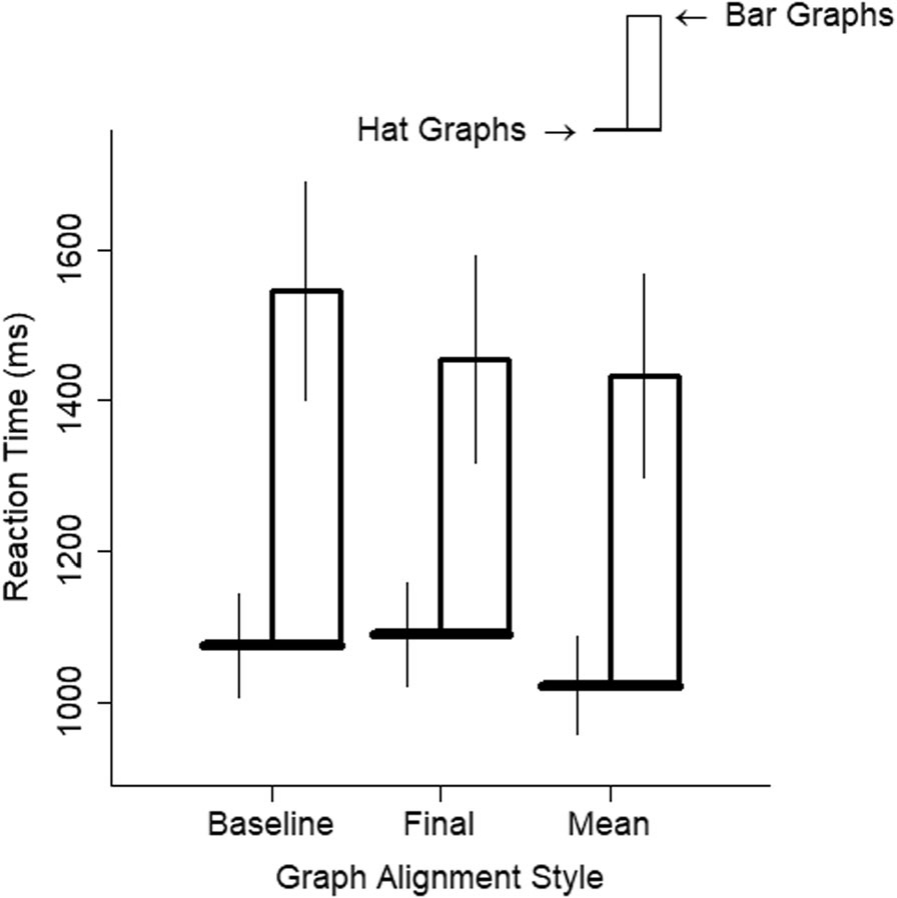
Reaction time (RT) is plotted as a function of graph alignment style and graph type for experiment 2. The brim of the hat corresponds to the RTs for the hat graph condition, and the top of the crown of the hat corresponds to RTs for the bar graph condition. The size of the crown indicates the difference in RTs between the two conditions. Error bars are ±1 SEM calculated from the linear mixed model

Reaction times were similar regardless of the alignment in the graph. For the comparison between aligned baseline scores and aligned final scores, the difference in RTs was very small, *d* = 0.12, *t* = 3.83, *p* < .001. This difference was similarly small for the comparison between aligned baseline scores and aligned mean scores, *d* = 0.15, *t* = 4.81, *p* < .001, and there was no difference between aligned final scores and aligned mean scores, *d* = 0.03, *t* = 0.98, *p* = .33. The interaction between graph type and aligned baseline versus final scores was also very small, *d* = 0.14, *t* = 3.72, *p* < .001. The interaction between graph type and aligned baseline versus mean scores was negligible, *d* = 0.05, *t* = 1.12, *p* = .26, as was the interaction between graph type and aligned final scores versus mean scores, *d* = 0.10, *t* = 2.16, *p* = .031. More importantly, the advantage of hat graphs was shown across all alignment styles, replicating some robustness to the advantage of hat graphs over bar graphs, *d*s ≥ 0.80, *p*s < .001 (see Fig. 9).

**Fig. 10:**
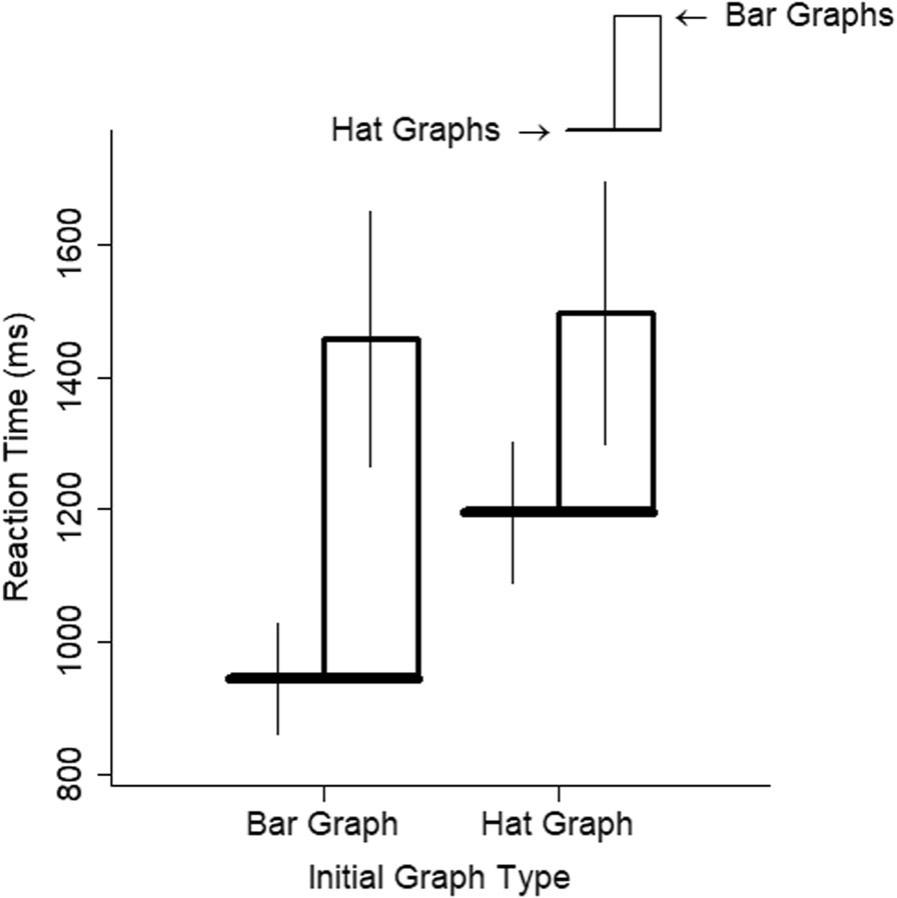
Reaction time (RT) is plotted as a function of graph type and start condition for experiment 2. The brim of the hat corresponds to the RTs for the hat graph condition, and the top of the crown of the hat corresponds to RTs for the bar graph condition. The size of the crown indicates the difference in RTs between the two conditions. Error bars are ±1 SEM based on estimates from the linear mixed model

Participants who started with bar graphs were somewhat faster with hat graphs relative to bar graphs compared with participants who started with hat graphs. The interaction between graph type and initial graph type showed a small-to-medium effect, *d* = 0.41, *t* = 2.25, *p* = .044 (see Fig. 10).

The results from experiment 2 replicate those from experiment 1 and show an advantage for the hat graphs over the bar graphs with respect to ease of processing differences across conditions, as shown by faster response times.

## Experiment 3

Experiments 1 and 2 show an advantage for hat graphs over bar graphs in that people were faster to identify the advertisement that produced the biggest increase in performance. Speed can be indicative of the ease with which a graph can be processed. However, there are other relevant components of graph comprehension including accuracy and biases.

One of the motivators for hat graphs as a replacement for bar graphs is that the hat graphs are not restricted to having the y-axis range start at zero. For bar graphs, the top of the bar and the length of the bar both signify the value for that condition. Consequently, bar graphs must start at zero to avoid having a conflict between the two indicators that could produce misleading impressions (Pandey et al., 2015; Pennington & Tuttle, 2009). This restriction does not apply to hat graphs because there is only the one indicator for the condition’s value. Thus, one of the benefits of hat graphs is to be able to control the range of the y-axis to better convey the magnitude of the effect. Small effects should look small and big effects should look big. One way to achieve this is to set the range of the y-axis to 1.5 SDs (Witt, in press). In experiment 3, hat graphs were plotted with this 1.5 SD range, whereas bar graphs were plotted such that the y-axis started at zero to empirically test whether this theoretical advantage of hat graphs leads to empirical benefits.

### Method

#### Participants

Twenty-three participants completed the experiment in exchange for course credit. The first 13 completed the experiment with the two graph types presented in separate blocks, and the last 10 completed the experiment with the two graph types intermixed having decided the latter is more akin to what people are likely to experience.

#### Stimuli and apparatus

**Fig. 11:**
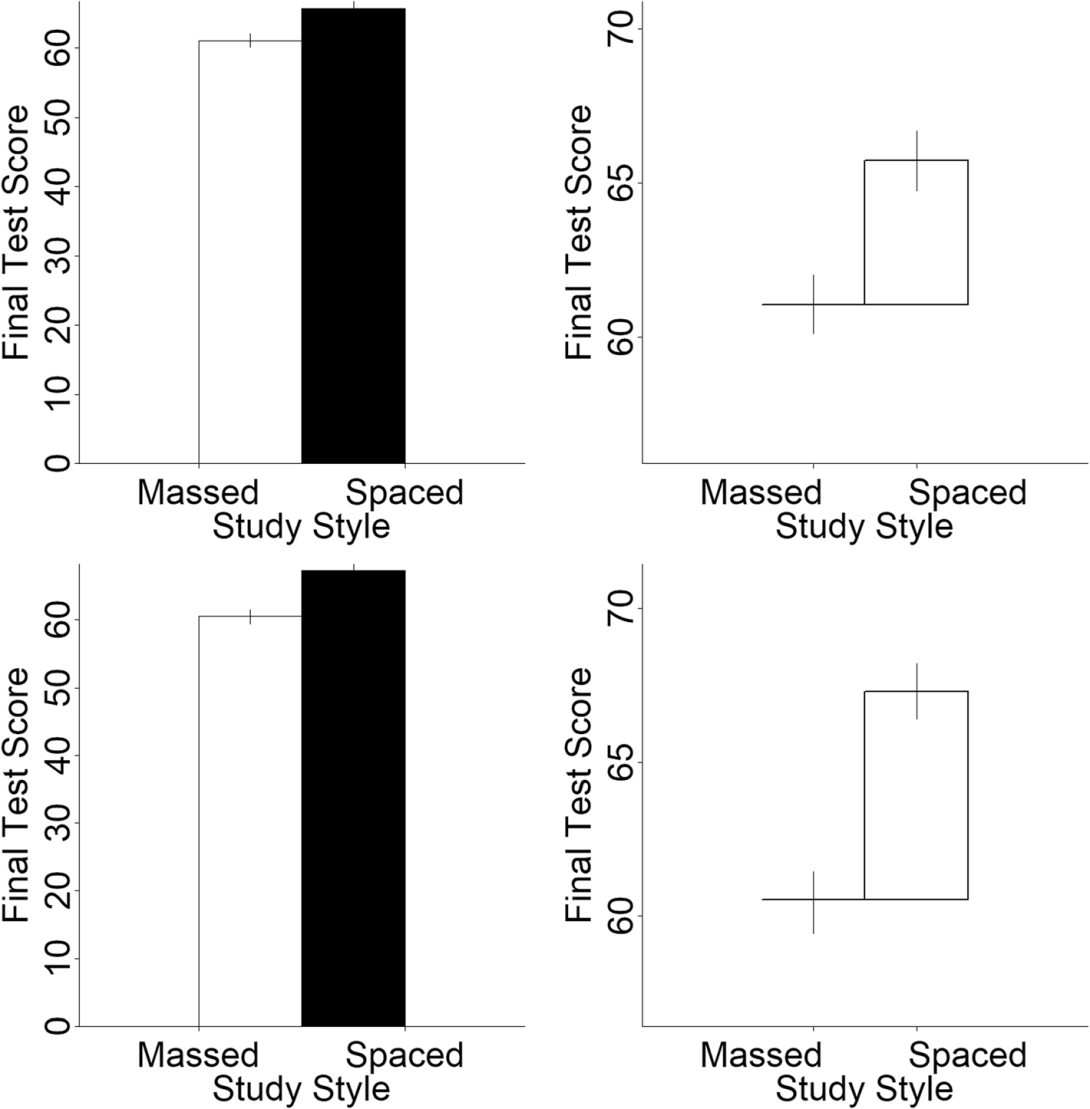
Sample stimuli for experiment 3. The top row shows the bar graph and the hat graph for the same set of data, which depicts a medium effect, and the bottom row shows each graph depicting the same big effect

The experimental set-up was the same as in experiments 1 and 2. The stimuli were 80 unique graphs, 40 of which were hat graphs and 40 were bar graphs. Each graph depicted two means based on simulated data. The data were simulated to mimic scores on a memory test from 0 to 100 after engaging in one of two study styles. Massed refers to studying everything at once, as in cramming just before the exam. Spaced refers to dispersing studying across time. The simulated mean for the massed study style was 60. The simulated mean for the spaced study style was set to one of four values (60, 63, 65, or 68). The simulated SD was 10 for both groups so the differences between the two groups correspond to four different effect sizes as measured with Cohen’s *d* (0, 0.3, 0.5, 0.8). These four values coincide with the naming conventions of a null, small, medium, and big effect. For each effect size, means for both groups were simulated based on a sample size of 100 per group, and for each effect size, simulations were conducted 10 times for a total of 40 unique data sets. The final effect size for each data set was compared to the intended effect size, and discarded and replaced if not within 0.05 SDs of each other. One bar graph and one hat graph was created for each data set, with error bars that corresponded to 95% confidence intervals (see Fig. 11). For the bar graph, the y-axis started at 0 and went to 4% beyond the top of the range necessary to see both error bars (as is the default in R). For the hat graph, the same restriction of having a baseline of zero does not apply. Therefore, the range of the y-axis was set to 1.5 SDs based on the recommendations of Witt (in press). The y-axis range was the grand mean of both groups minus 7.5 to the grand mean plus 7.5 for a total range of 15, which is 1.5 times the simulated SD of 10.

#### Procedure

Initial instructions explained the two study styles and that participants would make a judgment about whether study style affected final test performance. They were to judge whether study style had no effect, a small effect, a medium effect, or a big effect, and press the corresponding number (1–4, respectively). They were then given an overview of each type of graph showing what corresponded to the mean for each group and what the error bars signified. The two graph types were presented in different blocks for the first group of participants (*n* = 14), so instructions for each type of graph preceded that block. For the second group, the graph types were intermixed within block, so instructions for both graph types were presented at the beginning.

Each trial began with a blank screen with a fixation cross at the middle for 500 ms. Then the graph appeared and remained until participants estimated the magnitude of the effect depicted in the graph. There was no time limit and no feedback given. Responses were followed by a blank screen presented for 500 ms. For participants in the blocked condition, each block consisted of all 40 unique graphs for one type. Order within block was randomized. Participants completed two blocks with one graph type, then two blocks with the other graph type for a total of 160 trials. For participants in the intermixed condition, each block consisted of all 80 unique images (40 with each graph type, order was randomized), and participants completed three blocks for a total of 240 trials once it was determined that there would be enough time to complete all of these trials within the 30-min session.

### Results and discussion

The data were analyzed using a linear mixed model with Satterthwaite’s estimation for degrees of freedom. The dependent variable was estimated effect size, centered from the original scale of 1–4 by subtracting 3. One independent factor was depicted effect size. Although four effect sizes were used in the experiment, following Witt (in press), only the non-null effects were included in the analysis because there are differences in sensitivity when estimating between a null and a non-null effect compared with estimating across non-null effects, and it is the latter that is typically of greater interest. The three depicted effect sizes were converted to the same scale as the response and centered by subtracting 3 (− 1, 0, 1 for 0.3, 0.5, and 0.8, respectively). The other independent factors were graph type (bar graph and hat graph) and block type (blocked or intermixed). Depicted effect size and graph type were within-subject factors, and block type was a between-subject factor. Random effects for participant, including intercepts and main effects for each within-subject factors and their interaction, were included. These random coefficients were initially examined for outliers. One participant showed no sensitivity to effect size in either condition, and was excluded. The model was re-run but did not converge so the interaction term for the random effects was excluded to achieve convergence.

**Fig. 12:**
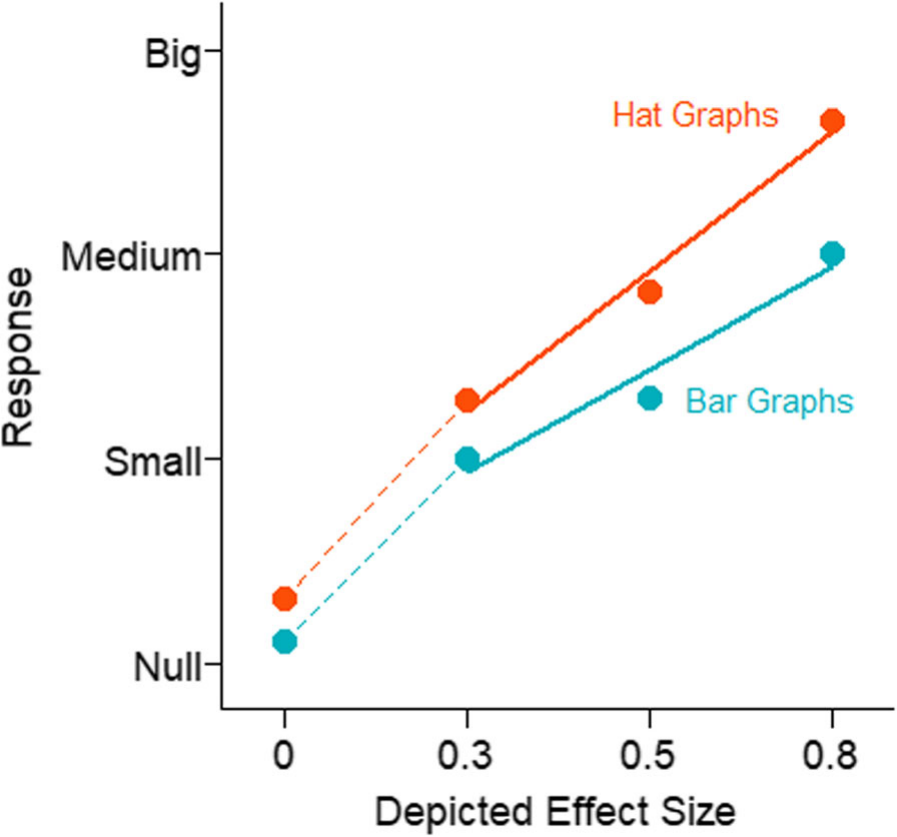
Mean response is plotted as a function of depicted effect size in the stimuli and graph type for experiment 3. Solid lines represent the mixed linear model coefficients. Steeper lines are indicative of better sensitivity. The dashed lines connect the mean estimated magnitude of null effects and small effects. The data from the depicted null effects were not included in the analysis

**Fig. 13:**
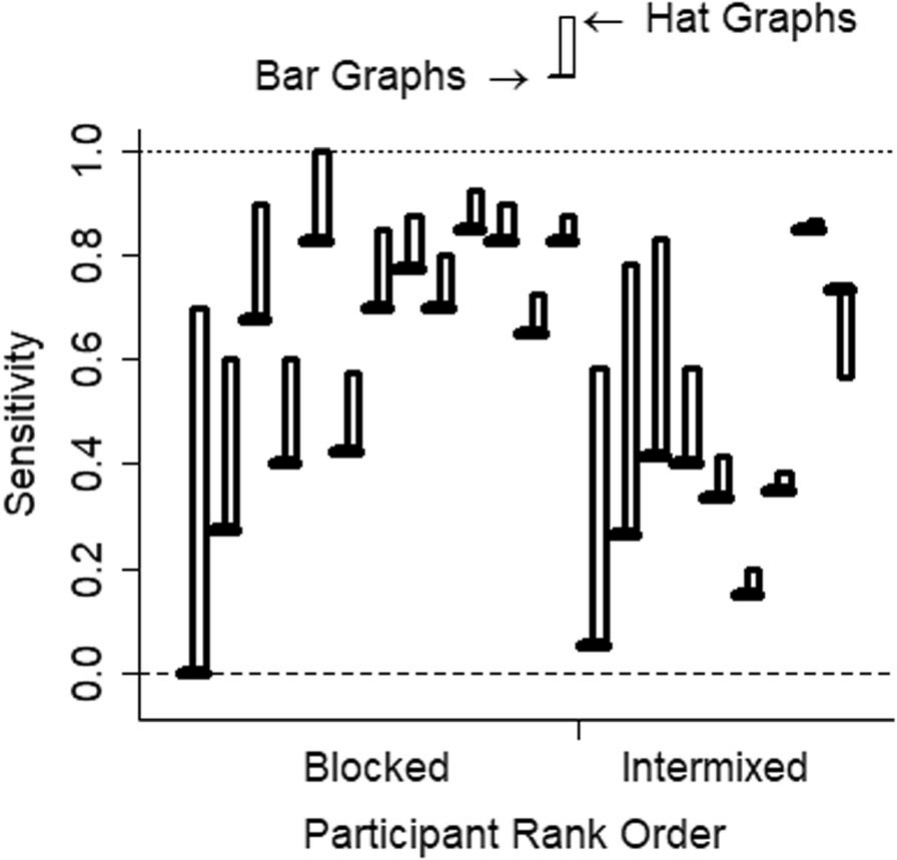
Sensitivity is plotted for each participant for each graph type. The brim of the hat corresponds to sensitivity with the bar graphs, and the crown corresponds to sensitivity with the hat graphs. Sensitivity was measured as the slope in linear regressions, and higher values correspond to better sensitivity. A slope of 1 corresponds to perfect sensitivity whereas a slope of 0 indicates no sensitivity. The brims correspond to the bar graph condition whereas the crowns correspond to the hat graph condition. Participants are grouped by design (blocked versus intermixed) then ranked with respect to how much more sensitive they were with the hat graphs compared with the bar graphs

With this experimental design, sensitivity is measured as the slope, and a slope of 1 indicates perfect sensitivity while a slope of 0 indicates no sensitivity. Sensitivity was 0.52 for the bar graphs (SE = 0.06) and was 0.70 (SE = 0.04) for the hat graphs. This shows a difference in sensitivity of 0.18, which corresponds to a 35% improvement in sensitivity for hat graphs with the standardized axes compared with bar graphs with the baseline at zero, *d* = 0.28, *t* = 6.28, *p* < .001, estimate = 0.18, SE = 0.03 (see Fig. 12). Separate linear models were run for each participant for each graph type. From these the slopes were extracted as the measure of sensitivity. Out of the 22 participants, 21 showed higher sensitivity with the hat graph than with the bar graph (see Fig. 13).

**Fig. 14:**
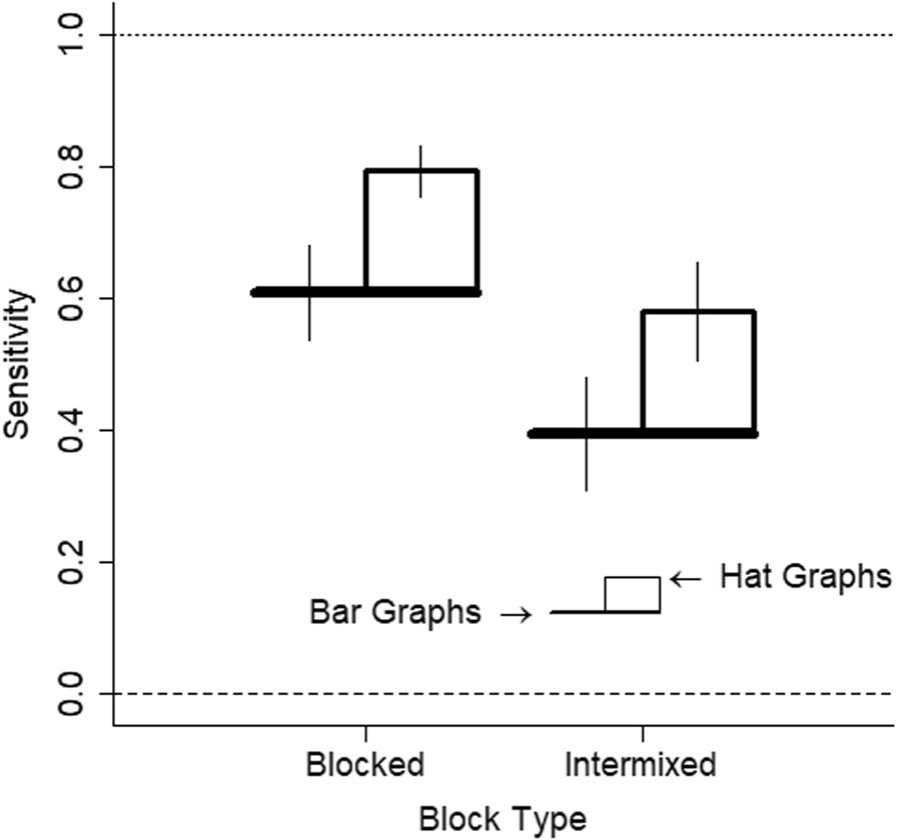
Sensitivity is potted as a function of block type and graph type. Sensitivity is measured as the slope. Higher scores indicate better sensitivity. Scores of 1 indicate perfect sensitivity and scores of 0 indicate no sensitivity. The brims correspond to the bar graph condition whereas the crowns correspond to the hat graph condition. Error bars are ±1 SEM calculated from the linear mixed models

There was a difference in sensitivity of 0.22 when the graphs were blocked than when they were intermixed, *d* = 0.33, *t* = 2.46, *p* = .022, estimate = 0.22, SE = 0.09. This makes sense given that participants did not have to rapidly switch between graph types to interpret graph size. The effect of block type on differences in sensitivity across graph types was negligible, *d* = 0, *t* = 0.01, *p* = .99 (see Fig. 14). In both cases, sensitivity was greater with the hat graphs than with the bar graphs (blocked, *d* = 0.30, *t* = 6.20, *p* < .001, estimate = 0.18, SE = 0.03; intermixed, *d* = 0.26, *t* = 6.49, *p* < .001, estimate = 0.19, SE = 0.03).

**Fig. 15:**
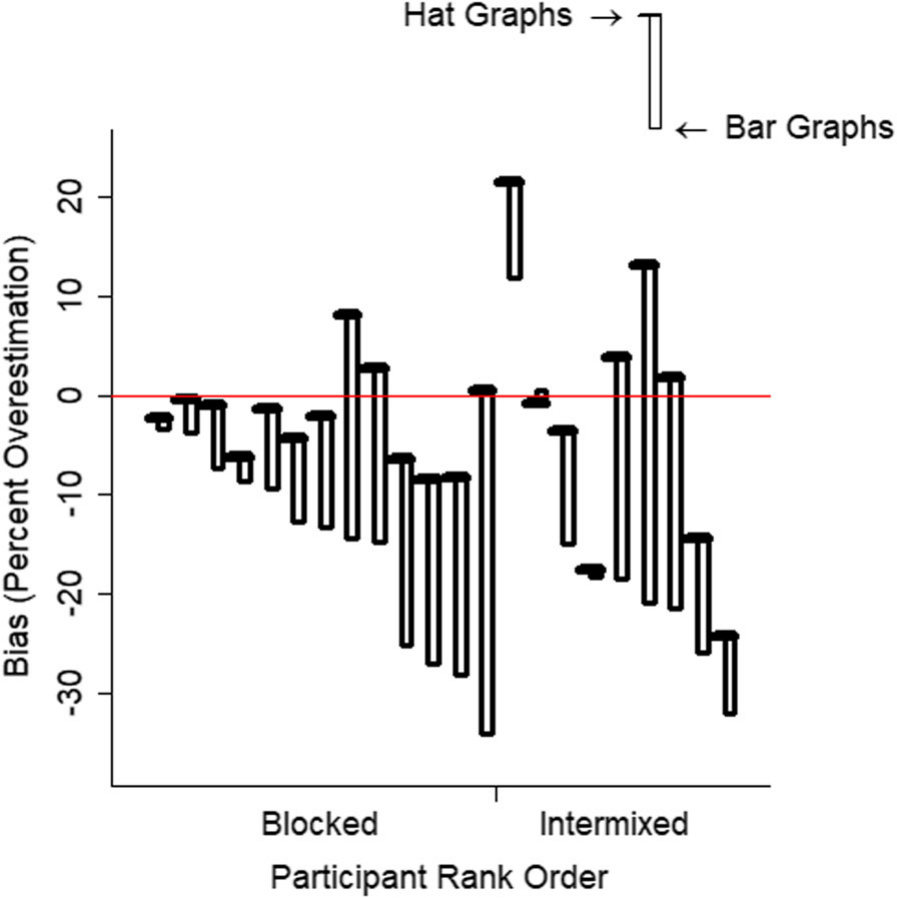
Bias in estimated effect size was calculated as a percent overestimation score. Positive values indicate a bias toward overestimation, and negative values indicate a bias toward underestimation. A score of 0 indicates no bias. Data are plotted for each participant, and participant is ordered based on design (blocked or intermixed) and then from largest to smallest bias with the bar graph. The brim corresponds to the hat graphs, and the edge of the crown corresponds to the bar graphs

In addition to sensitivity, the data also reveal biases associated with the various graph types. Bias is measured by the intercepts. Graph type had a medium effect on bias, *d* = 0.61, *t* = 4.33, *p* < .001, estimate = 0.40, SE = 0.09. Separate linear mixed models were run for each graph type to assess bias for each, and the intercepts were transformed into percent overestimation scores. When reading bar graphs, participants underestimated depicted effect size by 16%, *d* = 0.68, *t* = − 4.73, *p* < .001, estimate = − 0. 74, SE = 0.10. When reading hat graphs, the bias was 2% underestimation and was negligible, *d* = 0.11, *t* = − 0.79, *p* = .44, estimate = −0.07, SE = 0.09. Thus, not only do hat graphs improve sensitivity to effect size, they also reduce bias in estimating effect size relative to bar graphs because of their flexibility to allow the y-axis to start at a value other than zero. This leads to a bias to make effects appear smaller because bar graphs must start at zero. Figure 15 shows differences in bias for each participant.

It is also worth noting that error bars were included in both the hat graph and bar graph stimuli in this experiment. That the hat graphs still improved sensitivity and decreased bias despite the presence of the error bars further supports the claim of their advantage, at least when bar graphs are forced to a have a baseline at zero. One of the advantages of hat graphs is that they do not require the baseline to start at zero, whereas the rule for bar graphs is a baseline starting at zero. When following their respective rules, the hat graphs lead to increased sensitivity to the size of the effect depicted in the graph and reduced bias.

## Experiment 4

The rule is that bar graphs should have a baseline at zero. But rules are made to be broken, right? Currently, there is moderate agreement that bar charts should have the baseline at zero (Healy, 2019). Much of the debate takes place in blogs and on Twitter. For example, while Nathan Yau admits that most graphing rules have exceptions, he has not come across a worthwhile reason to break the baseline-at-zero rule for bar graphs (Yau, 2015). Stephen Few agrees (Few, 2019). In contrast, Kosslyn argues for maximizing compatibility between the visual impression of the display and the actual difference, and his specific example includes using a non-zero baseline with the bar graph (Kosslyn, 1994, p. 79). Others do not see the rule as specific to bar graphs given that non-zero baselines can lead to distortions in other kinds of graphs such as line graphs (Kosara, 2013; Skelton, 2018). Previously, I offered a way to avoid these distortions, at least in psychology and other behavioral sciences, by setting the range of the y-axis to equal 1.5 SDs (Witt, in press). This range increases sensitivity and minimizes bias relative to showing the minimal range or the full range. However, it means breaking the baseline-at-zero rule for bar graphs. Experiment 3 showed that sticking to the rule for bar graphs means that hat graphs have the advantage over bar graphs. Experiment 4 explored whether abandoning this rule would give bar graphs the advantage. Two other graph types were also included for comparison - boxplots and scatterplots.

### Method

#### Participants

Fifteen students participated in exchange for course credit.

#### Stimuli and apparatus

The stimuli were graphs based on data that were simulated from normal distributions. Data were simulated for two hypothetical groups with different study styles as in experiment 3. The mean for the spaced study group was 50, and the mean for the massed study group was 50, 47, 45, or 42, which corresponds to an effect size of *d* = 0, 0.3, 0.5, or 0.8. The SD was 10 for all simulations in both groups. The other factor was the number of samples drawn from each distribution. This number was determined based on achieving power of 0.80 or 0.95 to achieve the intended effect size. There were 10 repetitions for each effect size and for each power level. When the effect size was 0, power was based on intending to obtain one of the other effect sizes, and this varied across the 10 repetitions. Simulated data were checked to ensure that they were within 0.05 SDs of the intended effect size, otherwise the simulation was repeated until this condition was met. Altogether, there were 80 unique datasets.

Each simulated dataset was plotted four different ways for a total of 320 graphs. The massed style was the brim for the hat graphs and the spaced style was the crown. The first bar was white for bar graphs and represented the massed style, and the second bar was black and represented the spaced style. The y-axis was centered on the grand mean for both the bar and the hat graphs and extended 0.75 SDs in either direction. In addition, error bars representing ±1 SEM were also displayed. For the scatterplot, each data point was plotted as a separate open circle, and each was aligned along the x-axis based on study style (i.e., there was no jitter). The default parameters in R were used for the boxplot, so data points > 1.5 times the IQR were shown as open circles. The y-axis range was the default in R for both the scatterplot and the boxplot, which represented ±4% of the data range.

#### Procedure

Participants were given initial instructions that they would see data from two groups with different study styles and would have to indicate the size of the effect of study style on final test score. They were then given specific instructions on how to read each graph. The first two instructions pointed out the x-axis (study style) and the y-axis (final test score), which were the same for all graphs. Three images explained boxplot (the center line is the median score; the box represents 50% of all the scores in each group; the tails represent minimum and maximum values and the circles represent outliers that are likely not representative of the population). One image explained the scatterplot by stating that each point represents data from one participant. Three images explained bar graphs (the top of the box is the mean for each condition; the difference in heights is the difference between conditions; the error bars represent the precision of the estimate with longer lines meaning we are less certain of the accuracy of the mean). Three images explained the hat graphs in the same way as the bar graphs.

A fixation was present for 500 ms on each test trial, followed by a graph. The graph was visible until participants indicated their response by pressing 1, 2, 3, or 4 on the keyboard. A blank screen was presented for 500 ms. Each graph was presented once, and order was randomized for a total of 320 trials.

### Results and discussion

**Fig. 16:**
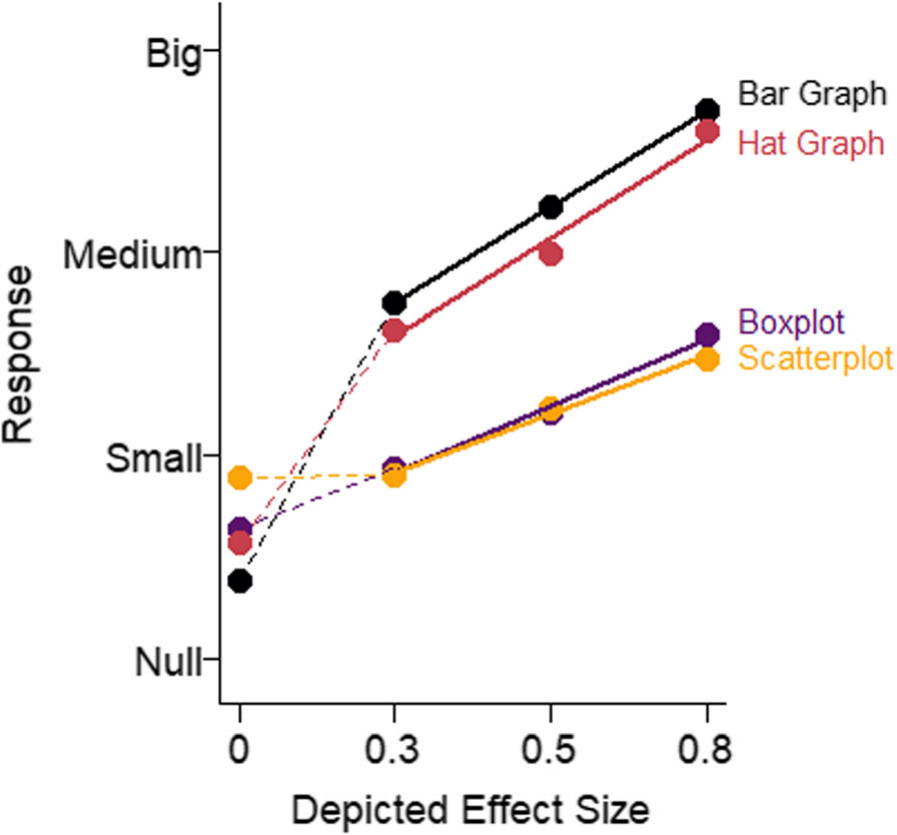
Mean response is plotted as a function of depicted effect size in the stimuli and graph type for experiment 4. Solid lines represent the mixed linear model coefficients. Steeper lines indicate better sensitivity. The dashed lines connect the mean estimated magnitude of null effects and small effects. The data from the depicted null effects were not included in the analysis

The data were analyzed as in experiment 3. As shown in Fig. 16, there were differences in sensitivity to effect size across graph types. However, these differences were primarily between graphs that showed means and SEMs (hat graphs and bar graphs) and graphs that showed more of the distribution (scatterplots and boxplots). Sensitivity for the hat graphs (estimate = 0.49, SE = 0.06) and sensitivity for the bar graphs (0.48, SE = 0.06) were equivalent, *d* = 0.01, *t* = 0.30, *p* = .76. When the bar graph had the same axes as the hat graph, there were similar levels of sensitivity. However, with respect to bias, the hat graph had a small advantage over the bar graph, *d* = 0.23, *t* = 4.33, *p* < .001. There was a small bias with the bar graph toward overestimating the size of the effect by 7% (*d* = 0.35, *t* = 2.15, *p* = .050). The bias was only 2% overestimation with the hat graph (*d* = 0.11, *t* = 0.65, *p* = .53). Even when playing by “hat graph rules” rather than “bar graph rules”, the bar graph was still not as good as the hat graph because it produced more bias in how the effects were interpreted.

Sensitivity was worse for the scatterplots and boxplots. These graphs depict the distributions beyond just the mean and SE, and both led to approximately 36% reduced sensitivity compared with the hat and bar graphs, *d*s = 0.23, *p*s < .001 (scatterplot, estimate = 0.29, SE = 0.05; boxplot, estimate = 0.33, SE = 0.04). The difference in sensitivity between the scatterplots and bar graphs was negligible, *d* = 0.06, *t* = 1.25, *p* = .212. Both scatterplots and boxplots produced a large bias toward underestimating the size of the effect depicted in the graph (scatterplot, 27% underestimation, *d* = 1.09, *t* = 8.46, *p* < .001; boxplot, 25% underestimation, *d* = 1.26, *t* = 10.56, *p* < .001). There was also a negligible difference in bias between the two plots, *d* = 0.06, *t* = 0.61, *p* = .55. Scatterplots and boxplots provide more information than hat graphs and bar graphs, but this additional information had two negative impacts on graph comprehension - decreased sensitivity and increased bias. The current data support the idea that less can be more. A potentially relevant factor is that boxplots may not have been familiar to our participants, but scatterplots are fairly common, and performance was similar for the two graph types.

## Experiment 5

Although the hat graphs and bar graphs lead to better sensitivity and worse bias than the boxplots and scatterplots, sensitivity was still far from ideal. Furthermore, all graphs except the hat graph led to bias in how the effects were interpreted. In this experiment, feedback was provided after each trial to determine whether people could quickly learn how to accurately read each type of graph.

### Method

The experiment was the same as in experiment 4 except that feedback was given on every trial. Specifically, the correct response was provided after participants made each response, regardless of the accuracy of their response. Eleven students participated in exchange for course credit.

### Results and discussion

**Fig. 17:**
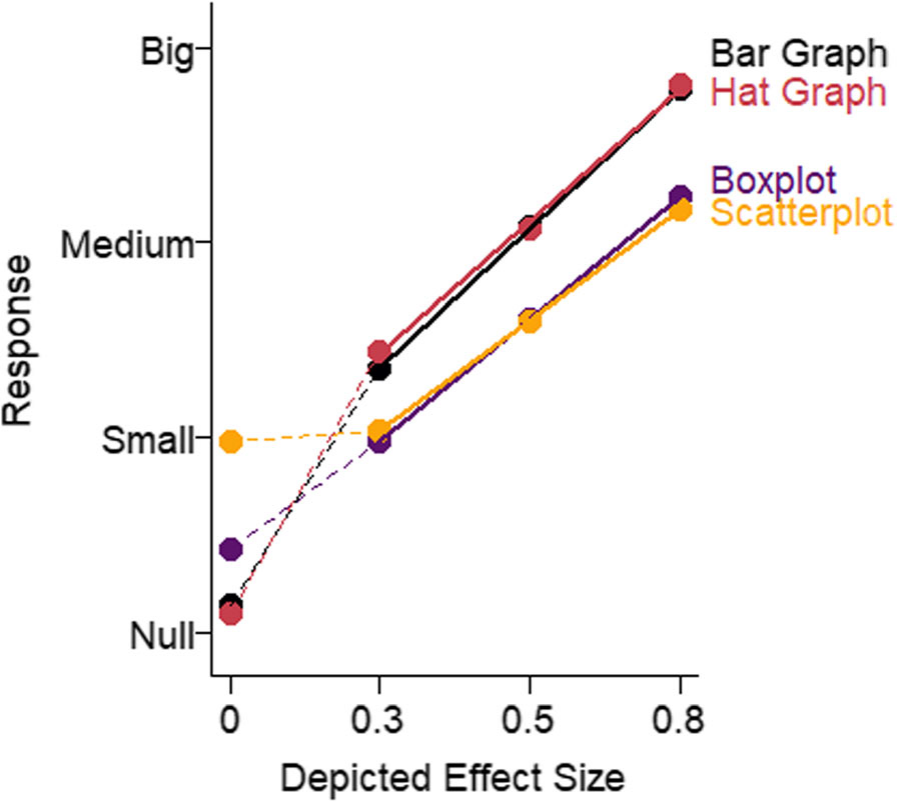
Mean response is plotted as a function of depicted effect size in the stimuli and graph type for experiment 5 for which feedback was provided. Solid lines represent the mixed linear model coefficients. Steeper lines indicate better sensitivity. The dashed lines connect the mean estimated magnitude of null effects and small effects. The data from the depicted null effects were not included in the analysis

**Fig. 18:**
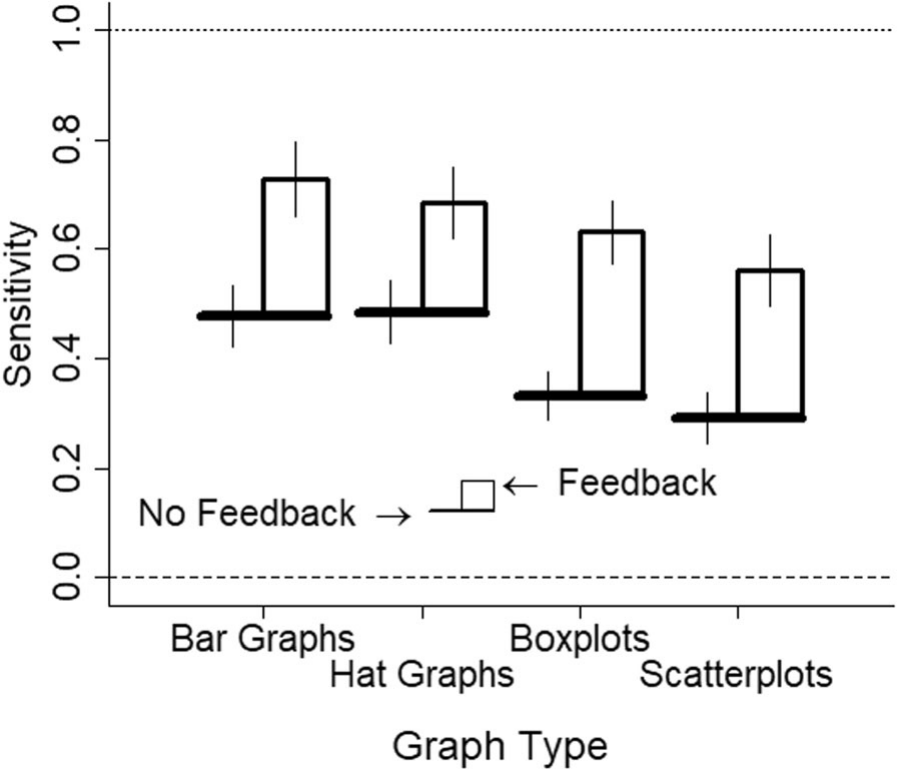
Sensitivity is potted as a function of graph type and whether there was no feedback (experiment 4) or there was feedback (experiment 5). Sensitivity is measured as the slope. Higher scores indicate better sensitivity. Scores of 1 indicate perfect sensitivity and scores of 0 indicate no sensitivity. The brims correspond to the no-feedback conditions and the crowns correspond to the feedback conditions. Error bars are ±1 SEM calculated from separate linear mixed models

**Fig. 19:**
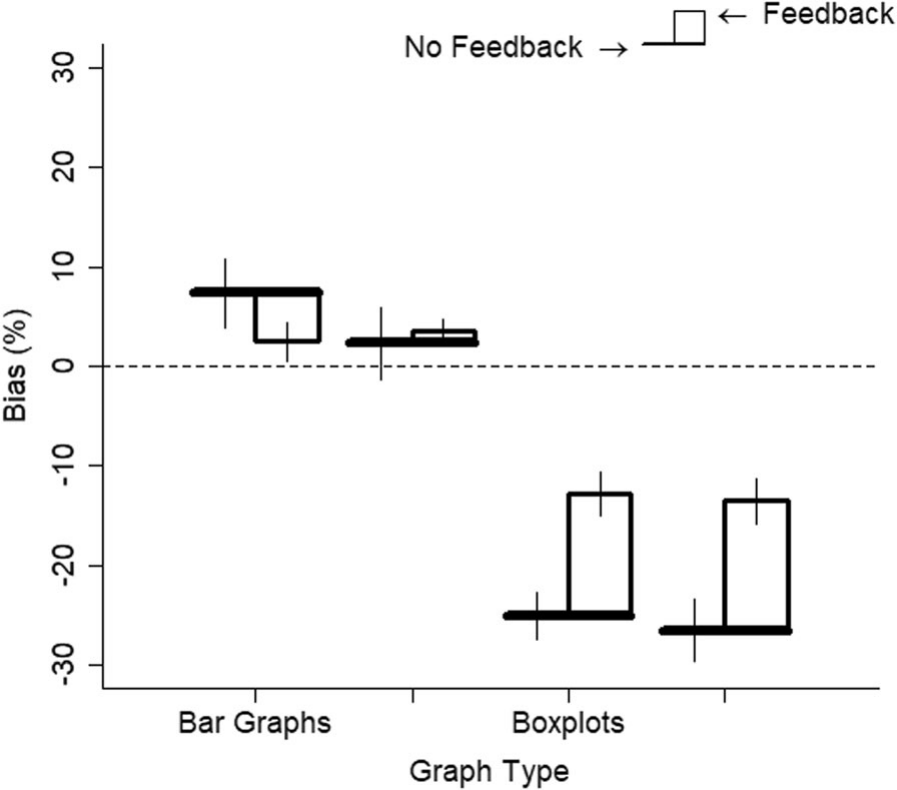
Bias (calculated as percent overestimation) is potted as a function of graph type and whether there was no feedback (experiment 4) or there was feedback (experiment 5). Positive scores indicate a bias toward overestimating the depicted effect, and negative scores indicate a bias toward underestimating the depicted effect. The brims correspond to the no-feedback conditions and the crowns correspond to the feedback conditions. Error bars are ±1 SEM calculated from separate linear mixed models

The data were analyzed as in experiment 4. There were negative slopes for one participant for two of the graph types and the participant was excluded. The results are shown in Fig. 17. All graphs showed good but attenuated sensitivity (hat graph, estimate = 0.69, SE = 0.07; bar graph, estimate = 0.73, SE = 0.07; boxplot, estimate = 0.63, SE = 0.06; scatterplot, estimate = 0.56, SE = 0.06). Compared with experiment 4, feedback improved performance for all the conditions, estimate = 0.20–0.30, SE = 0.07–0.09, *p*s < .05 (see Fig. 18). Feedback nearly equated performance across the graph types. The hat graphs and bar graphs were equivalent to each other, *d* = 0.06, *p* = .34. The difference between the hat graphs and bar graphs from the scatterplots and boxplots was either very small or negligible, *d*s = 0.08–0.19, *p*s = 0.006–0.27. However, the scatterplots and boxplots still led to a bias toward underestimatng the size of the depicted effect by 13%, *d*s = 0.43–0.49, *p*s < .001. This was reduced compared with experiment 4 but was nevertheless a small-to-medium effect (see Fig. 19). The bias was very small for the bar graphs and hat graphs, 2% and 4%, *d*s = 0.12 and 0.2, *p*s = .25 and .013, respectively.

The study shows similar performance across bar graphs and hat graphs when feedback was provided and when the rule of the zero baseline is ignored for the bar graphs. To the extent that the data follow a normal distribution that is well-described by the mean and SEM, graphs that show only these values better succeeded at communicating the magnitude of the difference between two groups. However, violations to the assumptions of a normal distribution such as skewness or outliers are not well-captured by hat graphs and bar graphs. Had the task for participants focused on these aspects of the data instead, we would expect better performance with the scatterplots and boxplots than with hat graphs or bar graphs.

## General discussion

Hat graphs were designed based on principles of graph design and Gestalt grouping to be a better alternative to bar graphs. Bar graphs have essentially remained unchanged since their inception in the late eighteenth century (Playfair, 1786). Hat graphs were designed as a simple but important modernization of the classic bar graph in an attempt to improve ease of graph comprehension. Two aspects of the benefits of hat graphs were explored here. One related to ease to ascertain differences represented in the graph. The other related to restrictions on the range of the y-axis and how eliminating these restrictions could improve sensitivity to and reduce bias of estimates of the magnitude of the effect depicted in the graph. Each will be discussed in turn.

Often the purpose of a graph is to invite comparison between two or more conditions. These comparisons are better served by maximizing proximity between the objects that represent each condition, but bar graphs have no inherent restrictions on the spacing between the bars, thereby allowing the bars to be spaced far apart and making the comparison more difficult. Hat graphs remedy this by using two of the strongest Gestalt principles of grouping - connectedness and proximity (Han, Humphreys, & Chen, 1999; Palmer & Rock, 1994; Wagemans et al., 2012; Wertheimer, 1912). For the hat graph, two conditions to be compared are placed adjacent to each other in a way that their components are connected. The visual system should therefore group the elements into a single perceptual object. Hat graphs necessitate this visual grouping, whereas bar graphs can have proximity and connectedness to help visually group objects, but it is optional and left to the designer’s choices.

Hat graphs also improve comparison across conditions because they make the difference between conditions an object itself, rather than the space between two objects as is the case with the bar graph. With bar graphs, each bar has an associated message flag signaling its height, and additional processes of interrogation are necessary to visually compare across heights (Pinker, 1990). With the hat graph, the difference is represented as an object. The height of this object, which represents the magnitude of the difference, has its own message flag signaling this value. Thus, the difference can be automatically extracted from the graph, thereby reducing the number of processing steps and improving ease of comprehension of the graph. The current experiments empirically tested this prediction by assessing the speed by which people could identify the biggest difference across conditions. Participants viewed graphs depicting attitude scores at a baseline test and at a final test across three or six advertisements. Their task was to indicate the advertisement that led to the biggest boost in attitude. Participants were almost 40% faster when the data were presented in hat graphs than when presented in bar graphs. Nearly all participants showed faster response times with the hat graphs than with the bar graphs. This result provides a good test of Pinker’s model of graph comprehension, and the outcomes are consistent with the model’s predictions.

Prior evidence showed that discrete elements (like in bar graphs) are more likely to be interpreted as a discrete effect (e.g. “males are taller than females”) whereas continuous lines (like in line plots) are more likely to be interpreted as a continuous effect even when such an interpretation is inappropriate (e.g. “the more male a person is, the taller he is”, Zacks & Tversky, 1999). Although not directly tested here, the hat graphs bear more of a resemblance to the discrete elements of the bar graph than to the continuous elements of the line graph. It is recommended that hat graphs be used instead of bar graphs for plotting discrete data, whereas line graphs continue to be used for continuous data.

Another graph design principle concerns the data-to-ink ratio (Tufte, 2001). According to this principle, redundant ink should be minimized within reason. For the bar graph, both the length of the two side lines and the position of the top of the bar are all redundant, so to minimize redundant ink, one could remove two of the three indicators (Tufte, 2001, p. 101). The hat graph has a higher data-to-ink ratio than the bar graph, which could be one of the factors contributing to its advantage. The hat graph still contains redundant elements (such as retaining the brim of the hat rather than just the crown). However, sometimes redundancy can be advantageous for reading graphs, so it is important to not take the data-to-ink ratio too far (Carswell, 1992; Gillan & Richman, 1994). Future studies could determine whether adding or deleting ink from hat graphs improves performance.

Perhaps more important than improving the data-to-ink ratio is that removing the redundancy in the bar graphs between the bar heights and the bar lengths eliminated the restriction of having the baseline of the y-axis at zero. Bar graphs should always have a zero baseline so that the impression given by the length of the bars is consistent with the values for each condition (e.g., Pandey et al., 2015; Pennington & Tuttle, 2009). Hat graphs do not have the same restriction because they do not contain the misleading element within bar graphs, namely bar length. The benefit of an unrestricted baseline is that the y-axis range can be set so that the visual impression of the size of the effect depicted in the graph aligns with the actual size of the effect. This setting improves sensitivity to and reduces bias to the magnitude of the effect (Witt, in press). Effect size in psychology is measured in terms of SDs and an effect of 0.8 SDs is considered big. Therefore, the range of the y-axis should be approximately 1.5 SDs (and never < 1 SD). Setting the y-axis in this way helps to maximize compatibility between the visual impression of the effect and the size of the effect. Small effects will look small and big effects will look big.

That this control over setting the range of the y-axis would be an advantage for hat graphs compared to bar graphs was tested in experiment 3. Participants viewed two means presented in a hat graph or in a bar graph, and had to judge whether the difference between the means showed a null, small, medium, or big effect. For the hat graph, the range of the y-axis was set at 1.5 SDs. For the bar graph, the baseline of the y-axis was zero and the maximum value was 4% higher than the 95% confidence interval of the largest mean. This coincides with the default for R. The measures of sensitivity and bias were the slopes and intercepts from linear regressions, respectively. The slopes were steeper, indicating heightened sensitivity, for the hat graphs relative to the bar graphs. Participants were better able to detect the magnitude of the effect depicted in the graphs when the data were plotted using hats rather than bars. The intercepts also showed a bias toward underestimating effect size with bar graphs but no bias with the hat graphs. Setting the baseline of the y-axis to zero can make effects appear smaller than they are, leading to misimpressions of effect size. With a y-axis range of 1.5 SDs, this bias is eliminated and participants can accurately discern effect size.

If the baseline-at-zero rule is ignored, bar graphs produced similar sensitivity to hat graphs, as shown in experiments 4 and 5. However, without feedback, the bar graph produced more bias toward overestimating the size of the effect compared with the hat graph, showing an advantage for the hat graph even when both had the same axis range. Thus, to produce the same sensitivity as the hat graph, the bar graph must be designed to violate the baseline-at-zero rule, which means that there are conflicting indicators (edge of the bar and length of the bar) that could produce misleading impressions, and the bar graph will lead to more bias.

Hat graphs were designed to clearly show the mean values for each condition and the difference between them. Another design alternative is to show only the difference scores as bars. This has the advantage of highlighting these differences but at the expense of eliminating data about the means for each condition. The use of hat graphs can solve this dilemma as both are plotted at the same time. One disadvantage of the hat graphs compared with plotting the difference scores is that the hats are unlikely to be perfectly aligned. The relative lengths of aligned bars are easier to discriminate than the relative lengths of misaligned bars (Cleveland & McGill, 1985). Thus, researchers will have to decide if the added benefit of more precise estimation of length outweighs the benefit of showing both the differences (as misaligned bars in the crown of the hat) and the condition means. Alternatively, numbers could be placed near the corresponding components of the hat.

Hat graphs have a number of limitations and raise future research questions. One question concerns the best way to add error bars. The graph stimuli used in experiment 3 and the graphs presented throughout this paper show one way to do so, but this format was not empirically tested. Another limitation is how best to signal when the direction of the hat is reversed from one condition to another (such as would be found in a crossed interaction). The figures throughout this paper were prepared by drawing the brim considerably thicker than the rest of the hat. Another option is to use a fill color for hats that are “upside-down,” although this would likely draw attention to these particular hats in ways that may not be best for the purpose of the graph. For example, if the goal is to find the advertisement that leads to the best boost in performance, highlighting advertisements that lead to decrements in performance by filling in those hats will distract readers to those hats instead of the ones showing the best improvement. A hat graph function in R has been provided at the osf.io link, and researchers can determine which options best suit their needs.

A critical limitation is that hat graphs are limited to 2 x *N* designs for which comparisons across pairs are the critical comparisons. It is unclear how to expand hat graphs to allow comparison across three or more conditions. Many reported studies involve designs with factors that have two levels, so the use of hat graphs can certainly be advantageous even if they are not generalizable to all research scenarios.

An unknown factor and potential limitation is that the current studies focused on a task in which participants had to identify differences between baseline and final scores. Other tasks could be to compare across baseline scores or across final scores. The nature of the task dictates which graph will be most appropriate (e.g., Gillan, Wickens, Hollands, & Carswell, 1998). Hat graphs proved to be more effective than bar graphs, boxplots, or scatterplots in finding differences and identifying their magnitude. Given the prevalence of research that reveals significant differences, hat graphs can better communicate these differences than bar graphs.

Hat graphs show that design principles based on the nature of cognitive processes (e.g., Gestalt grouping principles, message flags) can improve how researchers visualize and communicate their data. Relative to bar graphs, hat graphs improved ease of comprehension of a graph, as revealed by increased speed to compare differences across conditions, and as revealed by increased sensitivity and reduced bias to comprehend the magnitude of an effect depicted in the graph, particularly when bar graphs were forced to adhere to the rule that the baseline should always be set to zero.

## Data Availability

Data, scripts, and supplementary materials available at https://osf.io/khjb9/.
